# Efficacy of immunotherapy remained in patients with recurrent/metastatic non‐small‐cell lung cancer after surgery with or without postoperative thoracic radiotherapy: a bi‐center retrospective study

**DOI:** 10.1111/1759-7714.15384

**Published:** 2025-04-17

**Authors:** Yuqi Wu, Renda Li, Fengwei Tan, Jianzhong Cao, Nan Bi

**Affiliations:** ^1^ Department of Radiation Oncology, National Cancer Center/National Clinical Research Center for Cancer/Cancer Hospital Chinese Academy of Medical Sciences and Peking Union Medical College Beijing China; ^2^ Department of Thoracic Surgery, National Cancer Center/National Clinical Research Center for Cancer/Cancer Hospital Chinese Academy of Medical Sciences and Peking Union Medical College Beijing China; ^3^ Department of Radiation Oncology, Shanxi Province Cancer Hospital/ Shanxi Hospital Affiliated to Cancer Hospital Chinese Academy of Medical Sciences/Cancer Hospital Affiliated to Shanxi Medical University Taiyuan China

**Keywords:** efficacy, immunotherapy, non‐small cell lung cancer, surgery, thoracic radiotherapy

## Abstract

**Purpose:**

Since mediastinal lymph node dissection and radiotherapy (RT) have potential unclear impacts on pulmonary lymphatic system, this study aimed to assess the effectiveness of immune checkpoint inhibitors (ICIs) in recurrent/metastatic non‐small‐cell lung cancer (NSCLC) patients who previously received radical surgery with or without thoracic RT.

**Methods:**

Clinical data of patients who underwent pulmonary lobectomy with systematic lymphadenectomy (2000.1.1–2021.7.2) and received immunotherapy after progression were retrospectively analyzed. Efficacy was mainly evaluated based on progression‐free survival (PFS) from the start of the ICIs. Toxicity was defined as treatment discontinuation due to immune‐related adverse effects (irAEs).

**Results:**

Ninety‐five patients were enrolled in the final cohort and 30 (31.6%) patients received thoracic RT before ICI treatment. ICIs were administered as a first‐line systematic treatment in 52.6% of patients. The median follow‐up time was 14.7 months (95% confidence interval [CI] 13.3–18.7 months). The median PFS was 12.3 months (95% CI 8.5–36.6 months). Six (6.3%) patients had treatment suspended due to irAEs. Patients who received RT had comparable median PFS with the non‐RT group (17.0 months vs. 11.1 months, *p* = 0.16). Similar toxicity rates were observed. Similar mPFS were reported in the stage III subgroup (RT vs. non‐RT, 8.10 vs. 8.45 months, *p* = 0.86) or the subgroup treated by ICIs as primary systematic therapy (RT vs. non‐RT, 13.6 vs. 16.1 months, *p* = 0.45).

**Conclusions:**

ICIs remained effective in recurrent/metastatic NSCLC patients with radical surgery and RT did not significantly compromise therapeutic effects.

## Graphical abstract

At some point after undergoing radical surgery for lung cancer with or without thoracic radiation therapy afterwards, the patient experienced disease progression. Response was observed after receiving immunotherapy.

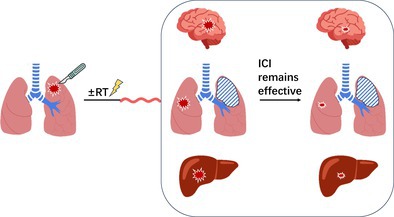

## INTRODUCTION

Lung cancer is the second most common cancer and remains the leading cause of death worldwide, according to GLOBOCAN estimation in 2020.^1^ According to histological type, over 80% of lung cancers were non‐small‐cell lung cancer (NSCLC).

In the last 10 years, monoclonal antibodies targeting programmed death protein‐1 (PD‐1) or its ligand (PD‐L1) have revolutionized the treatment of NSCLC and were considered standard care for advanced disease as first‐line or later therapy, supported by multiple large‐scale clinical trials. However, most of these trials included strictly naïve stage IV patients or did not elucidate. Only a few trials made it clear to include patients who had received definitive surgery or radiotherapy (RT) for primary disease or neoadjuvant or adjuvant therapy, such as CheckMate 057 and EMPOWER‐Lung 1.^2^, ^3^ Even in these trials, the following research mixed up progressive NSCLC after primary definitive treatment and de novo stage IV disease.

RT is a potential immune sensitizer that synergizes with immunotherapy in NSCLC.^4^, ^5^ Preclinical and clinical evidence suggested that the potential immunostimulatory mechanism of radiation includes^6^, ^7^, ^8^, ^9^ mediating cell death caused by DNA damage by stimulating the simulator of interferon genes‐nuclear factor kappa B (STING‐NFκB) signaling pathway, upregulating expression of major histocompatibility complex class‐I (MHC‐I), increasing expression of antigens and activating antigen‐presenting cells, which migrated to tumor‐draining lymph nodes (TDLNs) and then priming cytotoxic T lymphocytes, remodeling the tumor microenvironment, etc.

Theoretically, irradiation to the mediastinal lymphatic draining area can induce lymphopenia and may compromise the response to subsequential immunotherapy. In previous studies, radiation‐related lymphopenia was found to be associated with poorer prognosis in both postoperative and advanced NSCLC patients and significance increased with more intensive RT.^10^, ^11^, ^12^, ^13^ However, the influence of irradiation to TDLNs on the efficacy of immunotherapy afterwards was seldom discussed individually in NSCLC patients. A preclinical model found that addition of TDLN irradiation could impair survival when combined with immunotherapy.^14^

TDLNs act as both the first site of tumor metastasis and a pivotal organ for antitumor immunity in lung cancer. Whether thoracic RT could compromise therapeutic efficacy of ICIs in postoperative NSCLC patients is unclear. With complete resection of primary tumor and systematic mediastinal lymphadenectomy being the standard treatment pattern for early‐stage cancer in the past two decades and a considerable amount of TDLNs being removed, it is reasonable to explore the efficacy of immunotherapy in postoperative patients separately.^15^

This study aimed to assess the effectiveness and safety of immunotherapy in recurrent/metastatic NSCLC patients who previously received radical surgery with or without thoracic RT.

## MATERIALS AND METHODS

Consecutive patients at two institutions in China with operable NSCLC between January 1, 2000 and July 2, 2021 were identified. Inclusion criteria included aged over 18 years old with Eastern Cooperative Oncology Group performance status score 0–1, staged I–III NSCLC according to the 8th edition of American Joint Committee on Cancer (AJCC) staging system and underwent pulmonary lobectomy with systematic lymphadenectomy, including dissection at three or more mediastinal lymph node stations, and treated with PD‐1/PD‐L1 antibody after progression. Patients who had epidermal growth factor receptor or anaplastic lymphoma kinase genetic mutations or received target therapy, neoadjuvant or adjuvant immunotherapy, or thoracic radiation without mediastinal lymphatic drainage area were excluded. Thoracic RT ahead of immunotherapy delivered as adjuvant or salvage therapy for local‐regional recurrence were both eligible. Patients were further coded into RT and non‐RT groups according to whether or not they had postoperative RT.

Treatment response was evaluated according to Response Evaluation Criteria in Solid Tumors (RECIST) version 1.1 criteria based on computer tomography or magnetic resonance images. Participant follow‐up ended August 31, 2022. The primary endpoint was progression‐free survival (PFS), defined as the time from delivering the first dose of immunotherapy to either disease progression or death from any cause. Confirmation of disease progression was needed at least 4 weeks later. Overall survival (OS) was measured as the time between the administration of immunotherapy and death from all causes. The objective response rate (ORR) and the disease control rate (DCR) were defined as the proportion of patients with complete or partial response (CR/PR) and with CR/PR or stable disease (SD), respectively, based on RECIST version 1.1. Depth of response was defined as the nadir of the tumor response between the initiation of immunotherapy and the occurrence of progression or death events. The toxicity of immunotherapy was measured by the incidence of treatment discontinuation due to immune‐related adverse effects (irAEs). The severity of irAEs was graded using the 5th version of the National Cancer Institute's Common Terminology Criteria for Adverse Events version 5.0.

Baseline clinical characteristics as categorical variables were compared using Chi‐square test and Fisher's exact test, t test for continuous variables. Survival estimation was accomplished with Kaplan–Meier analysis and compared by log‐rank test. Covariates were evaluated using the Cox proportional hazard model. The inverse probability of treatment weighting (IPTW) was conducted to balance the confounding variables between subgroups, including age, gender, location of primary tumor, smoking history, pathology, stage, neoadjuvant and adjuvant chemotherapy history, PD‐1/PD‐L1 drug, and timing of immunotherapy. Results with *p* value less than 0.05 were considered significant. Analyses were all performed by R software version 4.1.3.

## RESULTS

Overall, 311 lung cancer patients treated with surgery and immunotherapy at two institutions in China were screened. A total of 95 NSCLC patients who had disease progression after surgery and received at least one cycle of immunotherapy were enrolled for final analysis (Supporting Information Figure S1).

The patients received surgery at a mean age of 60.3 years old, 84.2% were men, and 70 (73.7%) were current or former smokers. Forty‐seven patients (49.5%) were diagnosed with squamous cell carcinoma (SCC) and 44 (46.3%) were stage III lung cancer. Apart from standard lung lobectomy, 11.6% and 72.6% of patients received neoadjuvant and adjuvant platinum‐based chemotherapy. All patients had disease progression after surgery, with 25.3%, 46.3%, and 28.4% experiencing locoregional recurrence, distant metastasis, or combined failure. Thoracic RT was delivered in 14/95 as adjuvant therapy and 16/95 for locally recurrent disease, with the lymphatic draining area contained in the target volume during delineation. ICI was administered as first‐, second‐, or beyond‐line systematic treatment in 52.6% and 47.4% of patients who suffered from recurrent or metastatic disease afterwards. Immunotherapy was more frequently delivered in combination with chemotherapy or anti‐vascular endothelial growth factor (VEGF) therapy than as monotherapy (71.6% vs. 28.4%). PD‐1 inhibitors were chosen in most cases (97.9%), including pembrolizumab, nivolumab, camrelizumab, sintilimab, tislelizumab, toripalimab, nivolumab, geptanolimab, and penpulimab. Baseline clinical characteristics are presented in Table 1.

**TABLE 1:** Baseline characteristics of the overall cohort.

Characteristics		*N* (%)
Number of patients		95
Age (years)	Mean age (SD)	60.3 (8.8)
Gender	Male	80 (84.2)
Smoking history	Yes	70 (73.7)
Location	Upper	48 (50.5)
	Middle/lower	47 (49.5)
Pathology	SCC	48 (50.5)
	non‐SCC	47 (49.5)
Stage (AJCC 8th)	I–II	51 (53.7)
	III	44 (46.3)
NeoAC	Yes	11 (11.6)
AC	Yes	69 (72.6)
Radiotherapy	Yes	30 (46.2)
Timing of immunotherapy	First line	50 (52.6)
	Second line and beyond	45 (47.4)
Drug	PD‐1 inhibitor	2 (2.1)
	PD‐L1 inhibitor	93 (97.9)
Regimen	Monotherapy	27 (28.4)
	Combined therapy	68 (71.6)

*Abbreviations*: AC, adjuvant chemotherapy; AJCC, American Joint Committee on Cancer; NeoAC, neoadjuvant chemotherapy; PD‐1/PD‐L1, programmed death‐(ligand)1; SCC, squamous cell carcinoma; SD, standard deviation.

The median follow‐up time was 14.7 months (95% confidence interval [CI] 13.3–18.7 months) since the delivery of the ICI inhibitor. Fifty‐one patients had disease progression or death. The median PFS after the first dose of immune inhibitor was 12.3 months (95% CI 8.5–36.6 months) and 1‐year PFS was 51.9%. No predictive factor of PFS was found during univariate analysis (Supporting Information Table S1). In total, 12 deaths were observed. The median OS was not reached and the estimated 1‐year survival was 89.7%. Three patients were excluded from the analysis of DCR or ORR due to missing data. Among 92 evaluable patients, the DCR and ORR were 90.2% (95% CI 84.1%–96.3%) and 29.3% (95% CI 20.0%–38.7%).

In the entire cohort, six patients suffered from discontinuation of immunotherapy due to moderate or severe irAEs, including one Grade 3 (G3) fatigue, one G3 drug rash, one G3 immune‐related psoriasis, one Grade 2 (G2) increased alanine aminotransferase (ALT) and aspartate transaminase (AST), and two G2 pneumonitis. Four patients were retreated with ICIs, which were well tolerated (Supporting Inforamtion Table S2). No G4/5 adverse events were observed.

Patients were further divided into two groups, an RT group (*n* = 30) and a non‐RT group (*n* = 65). The RT group had a significantly higher proportion of stage III patients (70.0% vs. 35.4%, *p* = 0.003) and they were more likely to receive adjuvant chemotherapy (96.7% vs. 61.5%, *p* = 0.001). Immunotherapy alone was preferred when it was prescribed for patients with a previous history of thoracic RT (50.0% vs. 18.5%, *p* = 0.003). IPTW was then conducted, and all variables were well‐balanced between the two groups (Table 2).

**TABLE 2:** Baseline characteristics comparison between the RT and non‐RT groups.

Characteristics	Unmatched			IPTW		
	RT	Non‐RT	*p* value	RT	Non‐RT	*p* value
N	30	65				
Age, mean (SD) (years)	59.53 (8.15)	60.58 (9.17)	0.592	54.52 (8.16)	59.68 (9.88)	0.091
Gender (%)			0.454			0.399
Female	3 (10.0)	12 (18.5)		8 (7.8)	14 (15.1)	
Male	27 (90.0)	53 (81.5)		97 (92.2)	78 (84.9)	
Location (%)			0.301			0.127
Upper lobe	18 (60.0)	30 (46.2)		78 (74.1)	45 (49.3)	
Middle/lower lobe	12 (40.0)	35 (53.8)		27 (25.9)	47 (50.7)	
Smoking history (%)			0.762			0.433
No	9 (30.0)	16 (24.6)		17 (16.6)	24 (26.0)	
Yes	21 (70.0)	49 (75.4)		87 (83.4)	68 (74.0)	
Pathology (%)			0.771			0.349
SCC	16 (53.3)	31 (47.7)		41 (38.8)	42 (46.1)	
Non‐SCC	14 (46.7)	34 (52.3)		64 (61.2)	50 (53.9)	
Stage (%)			0.003			0.718
Stage I/II	9 (30.0)	42 (64.6)		64 (61.2)	50 (54.3)	
Stage III	21 (70.0)	23 (35.4)		41 (38.8)	42 (45.7)	
NeoAC (%)			1.000			0.302
No	27 (90.0)	57 (87.7)		99 (95.0)	82 (88.8)	
Yes	3 (10.0)	8 (12.3)		5 (5.0)	10 (11.2)	
AC (%)			0.001			0.600
No	1 (3.3)	25 (38.5)		43 (40.8)	26 (28.3)	
Yes	29 (96.7)	40 (61.5)		62 (59.2)	66 (71.7)	
Regimen (%)			0.003			0.776
Combined therapy	15 (50.0)	53 (81.5)		83 (79.0)	69 (75.5)	
Monotherapy	15 (50.0)	12 (18.5)		22 (21.0)	23 (24.5)	
Drug (%)			0.182			0.326
PD‐L1 inhibitor	2 (6.7)	0 (0.0)		2 (1.9)	0 (0.0)	
PD‐1 inhibitor	28 (93.3)	65 (100.0)		103 (98.1)	92 (100.0)	
Timing (%)			0.312			0.211
First line	13 (43.3)	37 (56.9)		31 (29.6)	47 (51.4)	
Second line or beyond	17 (56.7)	28 (43.1)		74 (70.4)	45 (48.6)	

Abbreviations: AC, adjuvant chemotherapy; IPTW, inverse probability of treatment weighting; NeoAC, neoadjuvant chemotherapy; PD‐1/PD‐L1, programmed death‐(ligand)1; RT, radiotherapy; SCC, squamous cell carcinoma; SD, standard deviation.

**FIGURE 1:**
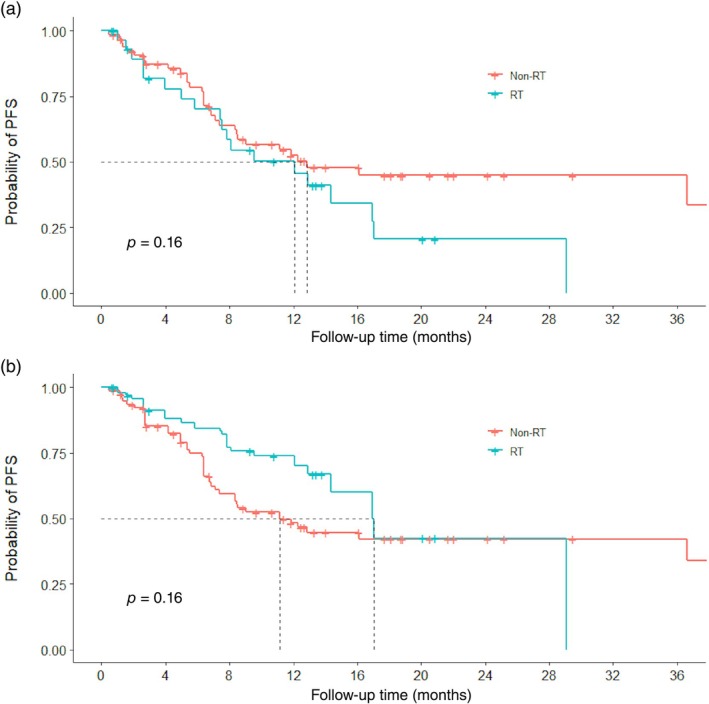
Kaplan–Meier estimates of progression‐free survival (PFS) in the non‐RT and RT groups before (a) and after (b) inverse probability of treatment weighting. RT, radiotherapy.

**FIGURE 2:**
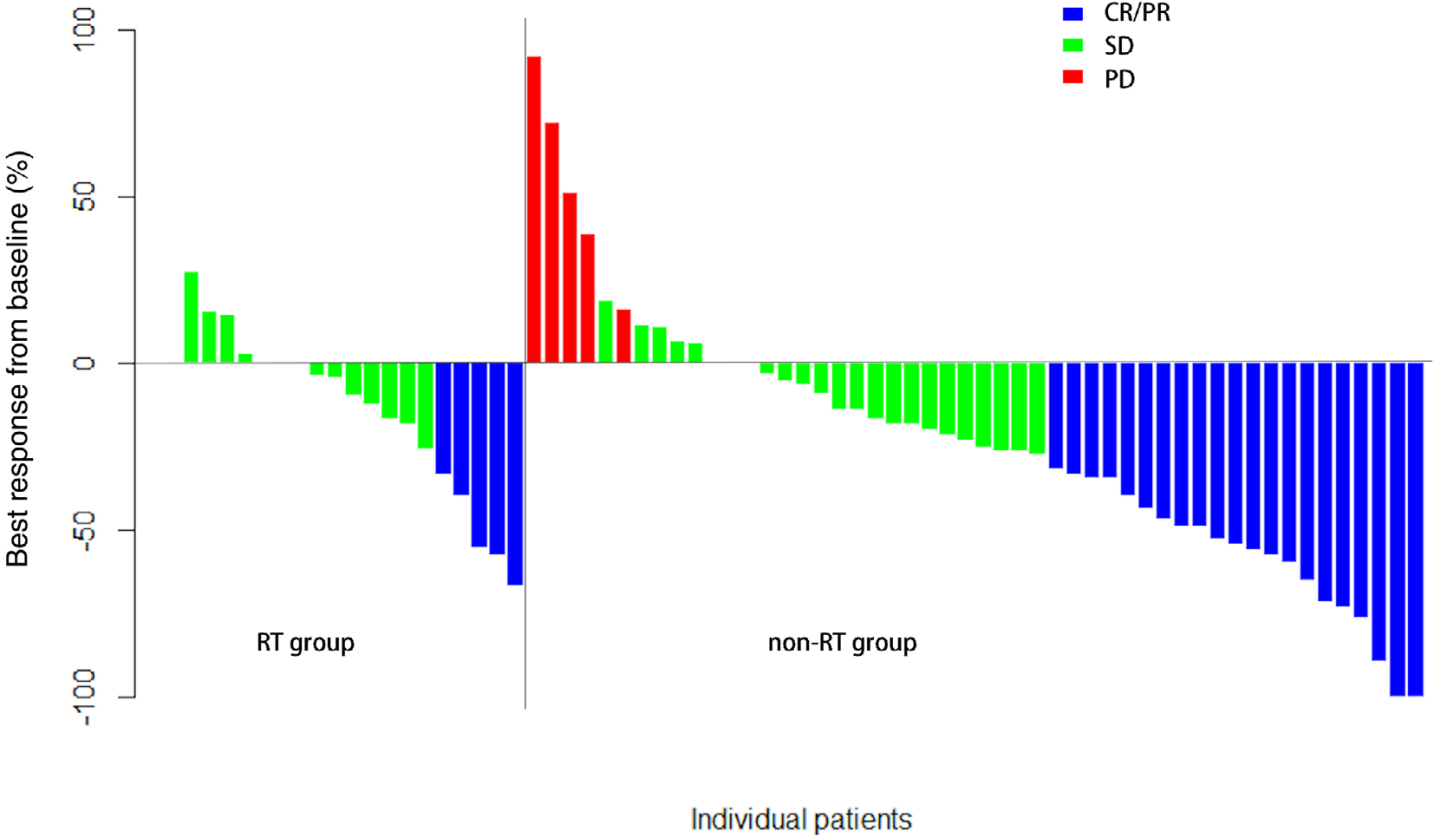
Depth of response in the RT and non‐RT groups. CR, complete response; PD, progressive disease; PR, partial response; RT, radiotherapy; SD, stable disease.

Comparisons of PFS between the RT and non‐RT groups pre‐ and post‐IPTW are shown in Figure 1. In the unmatched cohort, despite some poor prognostic factors observed in the RT group, the median PFS times were similar (12.1 months [95% CI 7.53–not available (NA)] in the RT group vs. 12.8 months [95% CI 8.47–NA] in the non‐RT group, *p* = 0.16, hazard ratio [HR] = 1.51 [95% CI 0.85–2.69]). No significant difference was found after IPTW (RT group 17.0 months, 95% CI 12.10–NA; non‐RT group 11.1 months, 95% CI 6.87–NA; *p* = 0.16). Higher DCR (96.3% vs 87.7%, *p* < 0.001) but lower ORR (18.5% vs 33.8%, *p* < 0.001) was observed in the RT group (Supporting Information Table S3). For the seven patients in the RT group who had only distant metastasis failure, the ORR was 28.6%. The depth of response of both groups is presented in Figure 2. No significant difference in toxicity was observed (Odds Ratio = 1.09, 95% CI 0.09–8.12).

**FIGURE 3:**
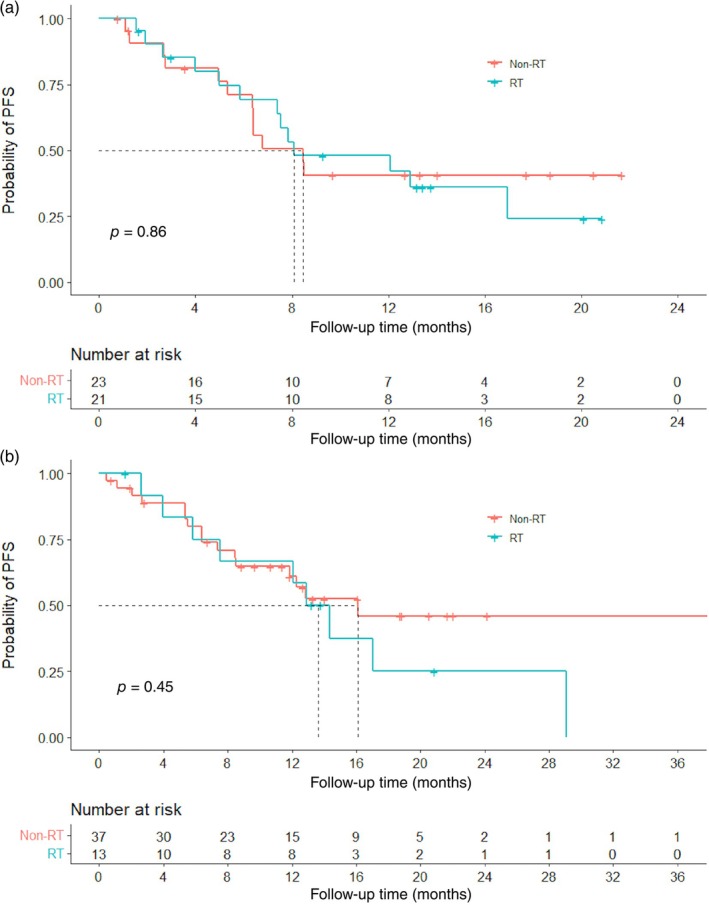
PFS comparisons of NSCLC patients with (blue) or without (red) previous thoracic radiotherapy in two subgroups: (a) stage III patients; (b) patients who received immunotherapy as the first‐line systemic therapy. NSCLC, non‐small cell lung cancer; PFS, progression‐free survival; RT, radiotherapy.

Further exploring analysis was done. Forty‐four patients had stage III NSCLC. Twenty‐one (47.7%) of them received thoracic RT and had similar median PFS to those in the non‐RT group (8.10 vs. 8.45 months, *p* = 0.86) (Figure 3). No significant difference in PFS was found in patients who received immunotherapy as first‐line systematic therapy, with or without previous thoracic RT (13.6 vs. 16.1 months, *p* = 0.45) (Figure 3).

## DISCUSSION

ICIs dramatically improve the survival of patients with advanced NSCLC and were recommended as first‐line systematic therapy and subsequent salvage therapy. Our study presents results exploring the impact of previous thoracic RT on subsequent anti‐PD‐1/PD‐L1 treatment in postoperative NSCLC patients.

The survival data in our research was numerically higher than that in previous results from classic phase III clinical trials (Supporting Information Table S4).^2^, ^3^, ^16^, ^17^, ^18^, ^19^, ^20^, ^21^, ^22^, ^23^, ^24^, ^25^, ^26^, ^27^, ^28^, ^29^, ^30^, ^31^, ^32^, ^33^, ^34^, ^35^ ICIs were considered effective in patients with previous definitive surgery, with the median PFS being 12.3 months. Median OS was not reached due to limited death events. More evidence was needed to find out whether distinguishing postoperative patients from advanced NSCLC patients can help to identify patients with better prognoses.

Patients treated with ICIs as first‐line had better PFS compared with those as  subsequent systematic therapy, but not significant. Numerous large‐scale randomized trials have shown that ICIs alone or with concurrent chemotherapy significantly prolonged survival compared with classic chemotherapy in SCC and non‐SCC NSCLC as first‐line systematic therapy (Supporting Information Table S4). PD‐1/PD‐L1 inhibitors were also assessed in previously treated patients, resulting in an improvement of PFS and OS. Median PFS and OS ranged from 2.5 to 2.8 months, and from 10.5 to 13.3 months in ICI monotherapy groups.

The synergistic effect of RT and immunotherapy was researched by multiple clinical trials. This sparked hope that metastatic NSCLC patients could convert an immunologically insensitive tumor into a sensitive lesion. Previous studies suggested that stereotacic body radiation therapy (SBRT) had great advantages over conventional RT combined with ICIs.^36^ A secondary analysis of the KEYNOTE‐001 trial discovered that previous extracranial radiation significantly increased median OS (11.6 months vs. 5.3 months, *p* = 0.024) and median PFS (6.3 months vs. 2.0 months, *p* = 0.0054) in patients with advanced NSCLC who received pembrolizumab treatment.^37^ In the PEMBRO‐RT trial,^38^ patients in the experimental arm had SBRT (24 Gy in three fractions) at any single site. In the M.D. Anderson Cancer Center (MDACC) phase I/II trial,^39^ only patients with lung or liver metastases that were amenable to either SBRT (50 Gy in four fractions) or hypofractionated RT (45 Gy in 15 fractions) were included. A pooled analysis of these two phase 1/2 trials enrolled 148 metastatic NSCLC patients.^40^ Seventy‐six patients were assigned pembrolizumab and 72 were assigned pembrolizumab plus RT. Radiation was given to at least one unirradiated lesion before or with pembrolizumab. Median PFS was 9.0 months in the combination therapy group versus 4.4 months in the pembrolizumab group (*p* = 0.045). However, in a recent study, metastatic NSCLC patients resistant to PD(L)‐1‐directed therapy were divided into three groups in a 1:1:1 ratio and received durvalumab plus tremelimumab alone or with the addition of low‐dose radiation every four cycles or hypofractionated RT only in the first cycle. No differences were found in overall response rates. The optimal radiation regimen remains to be determined. Practices in treating oligometastatic NSCLC patients could provide a reference, varying from 30 Gy in five fractions to 66 Gy in 30 fractions when mediastinum was involved. In addition, 45–55 Gy in 15 fractions and 60–66 Gy in 30 fractions were relatively common treatments.

However, whether the effect was maintained in advanced NSCLC patients after definitive surgery as in naïve stage IV NSCLC was unclear. Our findings focused on this population and found that a history of thoracic radiation had little influence on survival while patients receiving postoperative radiation therapy had more poor prognostic factors. A preclinical model compared tumor RT with or without radiation to TDLNs. The results showed that addition of TDLN irradiation could impair survival when combined with immunotherapy.^14^ Nevertheless, more prospective trials are needed for confirmation. Of note, we paid attention to thoracic RT with the mediastinal lymphatic drainage area included in the clinical target volume in this study, therefore conventional fraction radiation was widely delivered as adjuvant therapy and simultaneously integrated boosting (SIB) when patients had a local recurrence to meet normal tissue constraints.

Although no significant difference was found between the RT and non‐RT groups, patients in the RT group did show lower ORRs. Due to the relatively small sample size of this study, no statistical conclusion could be drawn, but we propose some possible reasons for the ORR trend. First, more lesions were defined as non‐evaluable, according to RECIST 1.1, in the RT group compared with the non‐RT group (22.2% vs. 13.8%). Hence, the efficiency of immunotherapy might be underestimated in RT group. Second, because of post‐RT fibrosis with poor blood supply, the efficacy of immunotherapy on locally recurrent tumors in the RT group might be restricted. When we focused on the seven patients in the RT group who had only distant metastasis failure, the ORR was better (28.6%) and comparable to 33.8% in the non‐RT group, therefore PFS might be a more appropriate index for evaluation.

The understanding of relationship between TDLNs and the efficacy of immunotherapy is insufficient. Haley du Bois et al. summarized data about TDLNs and found they dynamically evolved driven by inappropriate activation of immune cells and remodeling stroma to create an immune suppressive environment. TDLNs might act as a reservoir of tumor‐derived antigens and T cells, making them a potential target of treatment.^41^ Knowledge about the effects of RT on the function of TDLNs is limited. RT could increase cell death, enhance tumor antigen presentation, and subsequently enhance immune response. On the other hand, radiation‐induced lymphopenia had an adverse effect on patient survival. Multivariable analysis of 901 lung cancer patients revealed that severe lymphopenia (grade 3 or higher) was an independent poor‐prognostic factor of OS and was associated with mean lung dose.^42^ The effectiveness of ICIs remained no matter whether patients did or did not have thoracic RT in our study, but more experiments are necessary. Involved‐site RT and involved‐node RT deserve further study in the era of immunotherapy to maximize the efficacy of RT and minimize toxicity.

Concerns about the side effects of combination therapy were also raised. The safety profiles for PD‐1/PD‐L1 inhibitors were acceptable and thoracic RT did not significantly increase the incidence of treatment‐related toxicity in this study. No safety signals were newly identified. No severe (G4/5) irAEs occurred during treatment in this research. The well–well possibly caused by early regimen adjustment when mild adverse events were observed.

As well as the study's retrospective nature, limitations include the small number of patients and few death events. Absolute lymphocyte counts at baseline or before immunotherapy were not recorded. Details regarding the PD‐L1 status were lacking in this study due to a lack of records. However, the PD‐L1 expression level was associated with the effectiveness of ICI treatment^20^, ^21^, ^33^, ^37^ and could help in patient selection. For patients with PD‐L1 tumor proportion score (TPS) ≥50%, even ICIs alone could significantly prolong survival and were considered a better option than chemotherapy. For example, 305 naïve metastatic NSCLC patients with PD‐L1 TPS ≥50% were included in the KEYNOTE‐024 trial.^21^ Patients in the pembrolizumab group had better survival than patients in the chemotherapy group (median overall survival [mOS] 26.3 m vs. 13.4 m, HR = 0.62, 95% CI 0.48–0.81; median progression free survival [mPFS] 7.7 m vs. 5.5 m, HR = 0.50, 95% CI 0.39–0.65). Pembrolizumab, atezolizumab, and cemiplimab monotherapy is recommended as the preferred systematic therapy for PD‐L1 positive (≥50%) patients according to recently updated NCCN guidelines. We are looking forward to further detailed and well‐organized trials in the future.

In conclusion, immunotherapy remained effective in recurrent/metastatic NSCLC patients with upfront radical surgery and RT did not significantly compromise therapeutic effects.

## AUTHOR CONTRIBUTIONS

N.B. and F.T. contributed to the conception and design of the study. Y.W. and R.L. collected the necessary data. Y.W. and J.C. performed the statistical analysis. Y.W. wrote the first draft of the manuscript. R.L., F.T., J.C., and N.B. wrote sections of the manuscript. All authors contributed to the revision of the manuscript and approved the submitted version. All authors agreed to take responsibility for the contents.

## CONFLICT OF INTEREST STATEMENT

The authors declare that the research was conducted in the absence of any commercial or financial relationships that could be construed as a potential conflict of interest.

## Supporting information


**SUPPORTING INFORMATION FIGURE S1.** Flowchart of patient enrollment. NSCLC, non‐small‐cell lung cancer; EGFR, epidermal growth factor receptor; ALK, anaplastic lymphoma kinase; PD‐1/PD‐L1, programmed death‐(ligand)1.

**SUPPORTING INFORMATION TABLE S1.** Univariate analysis of PFS for the overall cohort.
**SUPPORTING INFORMATION TABLE S2.** Cases with treatment suspension due to irAEs.
**SUPPORTING INFORMATION TABLE S3.** DCR and ORR of RT and non‐RT groups.
**SUPPORTING INFORMATION TABLE S4.** List of classic phase 3 clinical trials on immunotherapy in advanced NSCLC.
