# Supplementary figures and images for: Efficacy of immunotherapy remained in patients with recurrent/metastatic non‐small‐cell lung cancer after surgery with or without postoperative thoracic radiotherapy: a bi‐center retrospective study
Source: Thorac Cancer. 2025 Apr 17;16(13):e15384. doi: 10.1111/1759-7714.15384 (PMC12245619; doi:10.1111/1759-7714.15384)

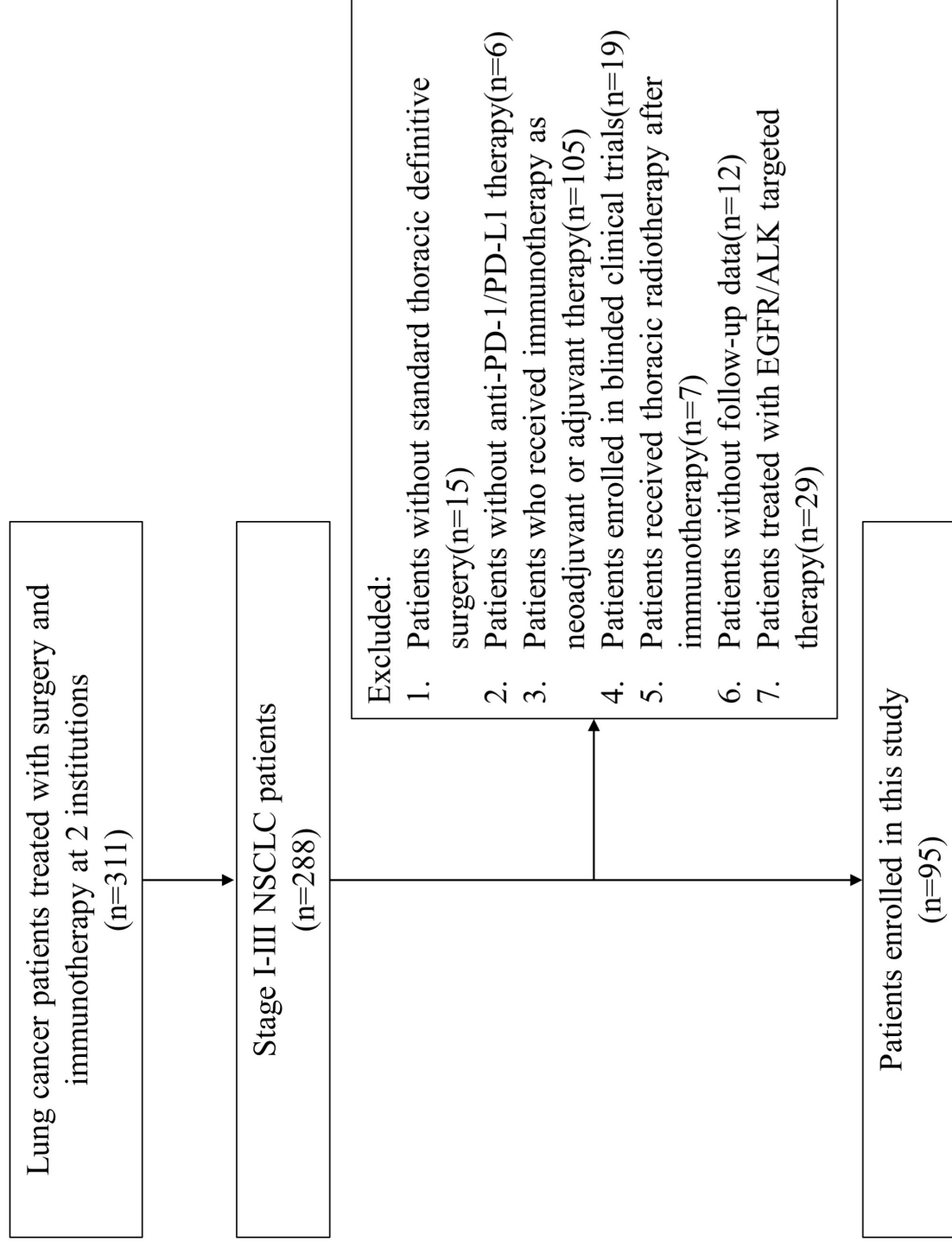

Supplement: Supplementary file 1 — SUPPORTING INFORMATION FIGURE S1. Flowchart of patient enrollment. NSCLC, non‐small‐cell lung cancer; EGFR, epidermal growth factor receptor; ALK, anaplastic lymphoma kinase; PD‐1/PD‐L1, programmed death‐(ligand)1. [file TCA-16-e15384-s001.pdf]
