# Supplementary material for: Efficacy of immunotherapy remained in patients with recurrent/metastatic non‐small‐cell lung cancer after surgery with or without postoperative thoracic radiotherapy: a bi‐center retrospective study
Source: Thorac Cancer. 2025 Apr 17;16(13):e15384. doi: 10.1111/1759-7714.15384 (PMC12245619; doi:10.1111/1759-7714.15384)
Supplement: Supplementary file 2 — SUPPORTING INFORMATION TABLE S1. Univariate analysis of PFS for the overall cohort. SUPPORTING INFORMATION TABLE S2. Cases with treatment suspension due to irAEs. SUPPORTING INFORMATION TABLE S3. DCR and ORR of RT and non‐RT groups. SUPPORTING INFORMATION TABLE S4. List of classic phase 3 clinical trials on immunotherapy in advanced NSCLC. [file TCA-16-e15384-s002.docx]

**Supplementary Table 1** Univariate analysis of PFS for the overall cohort.

| Variables | p-Value | Hazard Ratio(95%CI) |
| --- | --- | --- |
| Age | 0.138 | 1.03(0.99-1.06) |
| Gender | 0.177 | 1.74(0.78-3.89) |
| RT | 0.160 | 1.51(0.85-2.69) |
| Location | 0.176 | 1.47(0.84-2.56) |
| Smoke | 0.913 | 1.03(0.57-1.89) |
| Pathology | 0.858 | 1.05(0.60-1.85) |
| Stage III | 0.176 | 1.48(0.84-2.61) |
| NeoAC | 0.393 | 1.46(0.62-3.44) |
| AC | 0.476 | 1.27(0.66-2.43) |
| Regimen | 0.335 | 1.34(0.74-2.43) |
| Drug | 0.850 | 1.21(0.17-8.80) |
| Timing | 0.242 | 1.39(0.80-2.43) |

Abbreviations: PFS= progression-free survival. NeoAC= Neoadjuvant chemotherapy. AC=Adjuvant chemotherapy. RT=Radiotherapy.

**Supplementary Table 2** Cases with treatment suspension due to irAEs.

| ID | RT | Gender | Stage | Pathology | Regimen | irAEs | Retreated with ICI |
| --- | --- | --- | --- | --- | --- | --- | --- |
| 40 | No | Male | I | SCC | Monotherapy | G3 fatigue，G2 hypothyroidism, G1 dyspnea/ ALT increase | No |
| 50 | No | Male | III | SCC | Monotherapy | G2 ALT and AST increase | Yes |
| 56 | Yes | Female | III | Non-SCC | Combined therapy | G2 pneumonitis | Yes |
| 74 | Yes | Male | I | Non-SCC | Monotherapy | G2 pneumonitis | Yes |
| 88 | No | Male | I | Non-SCC | Combined therapy | G3 Drug rash, G2 pruritus | Yes |
| 95 | No | Male | III | SCC | Combined therapy | G3 Immune-related psoriasis, G2 pruritus | No |

Abbreviation: irAEs=Immune-related adverse events. SCC= squamous cell carcinoma; ICI= immune checkpoint inhibitor.; ALT = Alanine aminotransferase; AST=Aspartate aminotransferase.

**Supplementary Table 3** DCR and ORR of RT and non-RT group.

|  | RT | non-RT | p-value | p-value (adjusted by IPTW) |
| --- | --- | --- | --- | --- |
| N | 27 | 65 |  |  |
| DCR/n(%) | 26(96.3) | 57(87.7) | ＜0.001 | 0.003 |
| ORR/n(%) | 5(18.5) | 22(33.8) | ＜0.001 | 0.006 |

Abbreviation: DCR=disease control rate; ORR= objective response rate; IPTW=Inverse Probability of Treatment Weighting.

**Supplementary Table 4** List of classic phase 3 clinical trials on immunotherapy in advanced NSCLC.

| Trials | Population | Treatment(n) | mPFS/months | mOS/months | |
| --- | --- | --- | --- | --- | --- |
| First-line |  |  |  |  | |
| Checkmate 227[14] | PD-L1 ≥1% NSCLC | Nivolumab+ Ipilimumab (n=396) | - | 17.1 | |
| Checkmate 9LA[15] | NSCLC | Nivolumab+ Ipilimumab (n=361) |  |  | |
| Keynote 407[16] | sqNSCLC | Pembrolizumab+CT(n=278) | 6.4 | 15.9 | |
| Keynote 042[17] | PD-L1≥1% NSCLC | Pembrolizumab(n=637) | 5.4 | 16.7 | |
| Keynote 189[18] | non-sqNSCLC | Pembrolizumab+CT(n=410) | 9.0 | 22.0 | |
| Keynote 024[19] | PD-L1≥50% NSCLC | Pembrolizumab(n=154) | 7.7 | 26.3 | |
| IMpower 150[20] | non-sqNSCLC | ABCP(n=356) | 8.3 | 19.2 | |
| IMpower 130[21] | non-sqNSCLC | Atezolizumab+CT(n=451) | 7.0 | 18.6 | |
| IMpower 131[22] | sqNSCLC | Atezolizumab+CT(n=343) | 6.3 | 14.2 | |
| IMpower 132[23] | non-sqNSCLC | Atezolizumab+CT(n=292) | 7.6 | 17.5 | |
| MYSTIC[24] | PD-L1≥25% NSCLC | Durvalumab(n=163) | 4.7 | 16.3 | |
|  |  | Durvalumab+Tremelimumab(n=163) | 3.9 | 11.9 | |
| RATIONALE304[25] | non-sqNSCLC | Tislelizumab+CT(n=222) | 9.7 | - | |
| NCT03594747[26] | sqNSCLC | Tislelizumab+CT(n=120/119) | 7.6 | - | |
| ORIENT-11[27] | non-sqNSCLC | Sintilimab+CT(n=266) | 8.9 | 24.2 | |
| ORIENT-12[28] | sqNSCLC | Sintilimab+CT(n=179) | 5.5 | - | |
| EMPOWER-Lung1[3] | PD-L1≥50% NSCLC | Cemiplimab(n=283) | 8.2 | NR | |
| GEMSTONE-302[29] | NSCLC | Sugemalizmab+CT(n=320) | 9.0 | - | |
| Second-line or later | |  |  |  | |
| Checkmate017[30] | sqNSCLC | Nivolumab(n=131) | 3.5 | 9.2 | |
| Checkmate057[2] | non-sqNSCLC | Nivolumab(n=292) | 2.3 | 12.2 | |
| Keynote 010[31] | PD-L1 ≥1% NSCLC | Pembrolizumab(10mg/kg n=346) | 4.0 | 12.7 | |
| OAK[32] | NSCLC | Atezolizumab(n=425) | - | 13.8 | |
| ARCTIC[33] | PD-L1≥25% NSCLC | Durvalumab(n=62) | 3.8 | 11.7 |  |
|  | PD-L1＜25% NSCLC | Durvalumab+tremelimumab(n=174) | 3.5 | 11.5 | |

Abbreviation: PFS= progression-free survival; sqNSCLC=squamous non-small cell lung cancer;CT=Chemotherapy; ABCP= Atezolizumab +Bevacizumab+ Carboplatin+Paclitaxel.
